# Proanthocyanidin accumulation and transcriptional responses in the seed coat of cranberry beans (*Phaseolus vulgaris* L.) with different susceptibility to postharvest darkening

**DOI:** 10.1186/s12870-017-1037-z

**Published:** 2017-05-25

**Authors:** José A. Freixas Coutin, Seth Munholland, Anjali Silva, Sanjeena Subedi, Lewis Lukens, William L. Crosby, K. Peter Pauls, Gale G. Bozzo

**Affiliations:** 1 Department of Plant Agriculture, University of Guelph, 50 Stone Rd E., Guelph, ON N1G 2W1 Canada; 2 Department of Mathematics and Statistics, University of Guelph, 50 Stone Rd E., Guelph, ON N1G 2W1 Canada; 3 Department of Biological Sciences, University of Windsor, 401 Sunset Ave, Windsor, ON N9B 3P4 Canada; 4 Present address: Department of Mathematical Sciences, Binghamton University (State University of New York), 4440 Vestal Parkway E., Binghamton, New York 13902 USA

**Keywords:** Anthocyanidin reductase, *Phaseolus vulgaris*, Proanthocyanidin, RNA-seq, Seed coat darkening, Transcriptome

## Abstract

**Background:**

Edible dry beans (*Phaseolus vulgaris* L.) that darken during postharvest storage are graded lower and are less marketable than their non-darkened counterparts. Seed coat darkening in susceptible genotypes is dependent upon the availability of proanthocyanidins, and their subsequent oxidation to reactive quinones. Mature cranberry beans lacking this postharvest darkening trait tend to be proanthocyanidin-deficient, although the underlying molecular and biochemical determinants for this metabolic phenomenon are unknown.

**Results:**

Seed coat proanthocyanidin levels increased with plant maturation in a darkening-susceptible cranberry bean recombinant inbred line (RIL), whereas these metabolites were absent in seeds of the non-darkening RIL plants. RNA sequencing (RNA-seq) analysis was used to monitor changes in the seed coat transcriptome as a function of bean development, where transcript levels were measured as fragments per kilobase of exon per million fragments mapped. A total of 1336 genes were differentially expressed between darkening and non-darkening cranberry bean RILs. Structural and regulatory genes of the proanthocyanidin biosynthesis pathway were upregulated in seed coats of the darkening RIL. A principal component analysis determined that changes in transcript levels for two genes of unknown function and three proanthocyanidin biosynthesis genes, *FLAVANONE 3-HYDROXYLASE 1*, *DIHYDROFLAVONOL 4-REDUCTASE 1* and *ANTHOCYANIDIN REDUCTASE 1* (*PvANR1*) were highly correlated with proanthocyanidin accumulation in seed coats of the darkening-susceptible cranberry bean RIL. HPLC-DAD analysis revealed that in vitro activity of a recombinant *Pv*ANR1 was NADPH-dependent and assays containing cyanidin yielded epicatechin and catechin; high cyanidin substrate levels inhibited the formation of both of these products.

**Conclusion:**

Proanthocyanidin oxidation is a pre-requisite for postharvest-related seed coat darkening in dicotyledonous seeds. In model plant species, the accumulation of proanthocyanidins is dependent upon upregulation of biosynthetic genes. In this study, proanthocyanidin production in cranberry bean seed coats was strongly associated with an increase in *PvANR1* transcripts during seed maturation. In the presence of NADPH, *Pv*ANR1 converted the physiologically relevant substrate cyanidin to epicatechin and catechin.

**Electronic supplementary material:**

The online version of this article (doi:10.1186/s12870-017-1037-z) contains supplementary material, which is available to authorized users.

## Background

Edible dry bean or common bean (*Phaseolus vulgaris* L.) is one of the most highly cultivated legumes, and is a primary source of dietary protein, fiber and vitamins in developing nations. In 2014, 25.1 million tonnes of edible dry bean were produced worldwide with the highest cultivation occurring in India, Myanmar, Brazil, United States and Mexico [1]. There is evidence for two centers of domestication for *P. vulgaris*, specifically that of small seeded beans in Mexico (Mesoamerican) and large seeded beans in the South American Andes [2, 3]. Although, Andean cultivars (e.g., cranberry bean) are genetically distinct from Mesoamerican cultivars (e.g., pinto) [4], both are susceptible to postharvest-related seed coat darkening [5, 6].

At harvest, cranberry beans are characterized by the presence of red-coloured mottling on a cream coloured seed coat. The light background colour is transformed into a beige/brown colour with postharvest handling [5, 6]. Similarly, the beige background of pinto beans is susceptible to postharvest darkening [6–8]. Typically, seed coat darkening is promoted by light, humidity, atmospheric O_2_, and high temperatures during storage, as well as high moisture content in seeds [6, 9, 10]. In pinto bean, postharvest-related seed coat darkening is controlled by the presence of one dominant *J* allele, whereas seeds of homozygous recessive (*jj*) plants do not darken [6]. Control of postharvest-related seed darkening is an economically important issue as it is one of the factors that can lead to reduced quality and an overall lower grade for the dry bean market [11]. In addition, darkened seed coats tends to be associated with a hard-to-cook trait [12, 13]. To date, the biochemical and molecular factors underlying the darkening of cranberry beans during postharvest storage remain unknown.

**Fig. 1:**
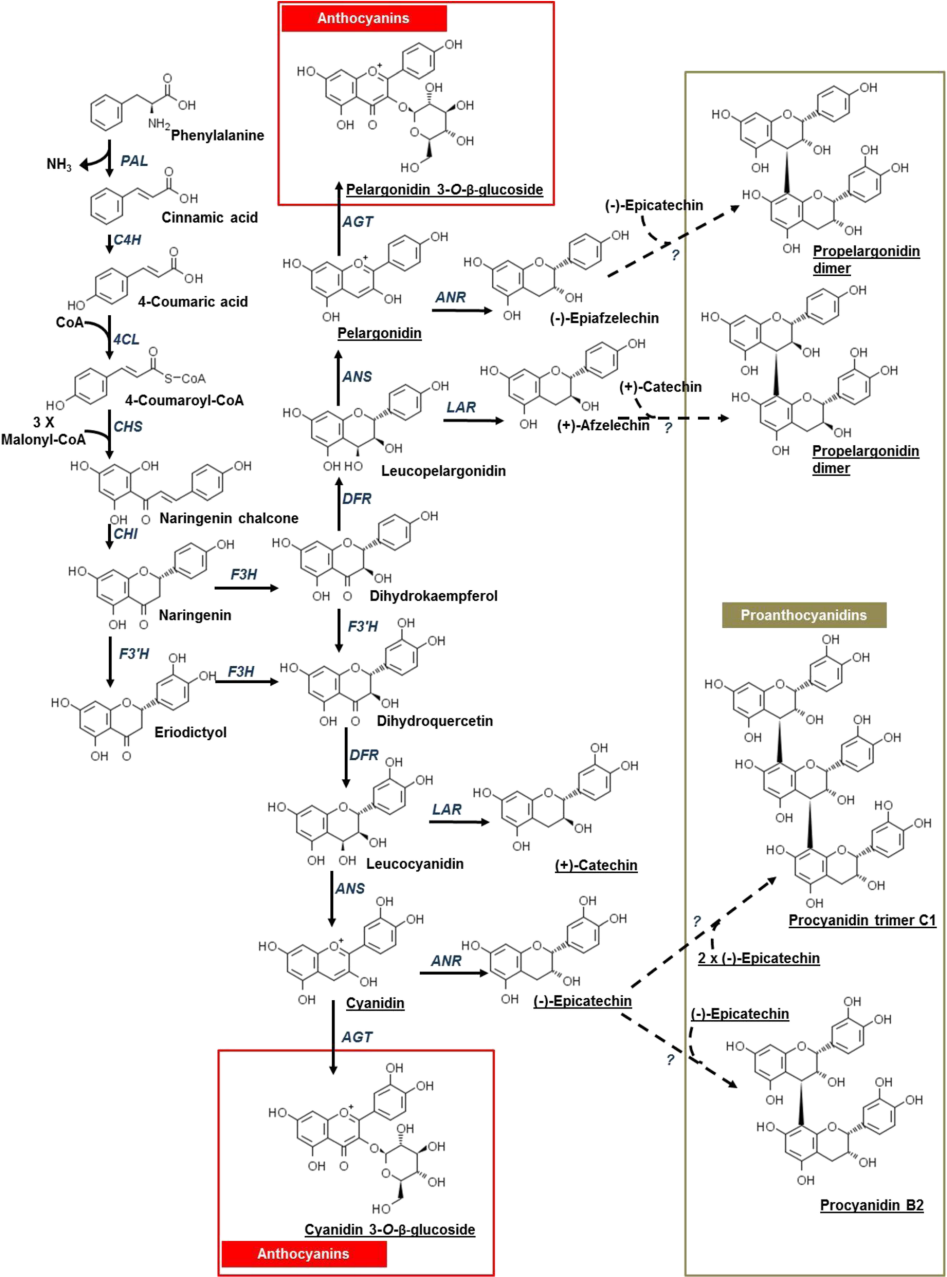
Proposed model of the proanthocyanidin biosynthesis pathway in cranberry bean seed coats. The proposed biosynthetic genes are based on information that is available for Arabidopsis and *M. truncatula* [17, 20, 22–24]. Structures corresponding to underlined anthocyanins, flavan-3-ols, and proanthocyanidins are based on HPLC-MS metabolite data described by Chen et al. [5, 41]. Gene abbreviations include: *ANR*, *ANTHOCYANIDIN REDUCTASE*; *ANS*, *ANTHOCYANIDIN SYNTHASE*; *AGT*, *URIDINE DIPHOSPHATE-GLUCOSE: ANTHOCYANIDIN 3-O-GLUCOSYLTRANSFERASE*; *CHS*, *CHALCONE SYNTHASE*; *CHI*, *CHALCONE ISOMERASE*; *C4H*, *CINNAMATE 4-HYDROXYLASE*; *4CL*, *4-COUMAROYL:COENZYME A LIGASE*; *DFR*, *DIHYDROFLAVONOL 4-REDUCTASE*; *F3′H*, *FLAVONOID 3′-HYDROXYLASE*; *F3H*, *FLAVANONE 3-HYDROXYLASE*; *LAR*, *LEUCOANTHOCYANIDIN REDUCTASE*; *PAL*, *PHENYLALANINE AMMONIA LYASE*

In legume seeds, proanthocyanidins accumulate within the endothelium of the seed coat [14, 15]. Their oxidation to reactive quinones promotes an interaction with proteins, culminating in brown deposits within this cell layer, including in pinto bean cultivars that are susceptible to seed coat darkening [7, 15]. Thus, seed coat darkening in legumes (e.g., dry bean, pea and soybean) is associated with the availability of proanthocyanidins, and similar phenomena occur amongst members of the Brassicaceae, including the model plant *Arabidopsis thaliana* [7, 16–21]. Proanthocyanidins (otherwise known as condensed tannins) are oligomers or polymers of flavan-3-ols (e.g., catechin and epicatechin) which are derived from the flavonoid biosynthesis pathway [22] (Fig. 1). Proanthocyanidin metabolism is well described for *Medicago truncatula*, *Vitis vinifera* and Arabidopsis. Moreover, the availability of a number of Arabidopsis pale seed or *TRANSPARENT TESTA* (*TT*) mutants has facilitated the elucidation of structural and regulatory steps that are functionally relevant for this pathway [23]. In Arabidopsis, proanthocyanidin biosynthesis gene transcripts are co-ordinately regulated and accumulate with seed development, reaching maximal levels at the mid to late torpedo stage of embryogenesis [24]. By contrast, gene expression for this pathway is highest at early stages of pea seed development, and in advance of proanthocyanidin accumulation in seed coats [19].

Proanthocyanidins are metabolically derived from phenylalanine in a series of steps catalysed by enzymes encoded by early biosynthesis genes; specifically: *PHENYLALANINE AMMONIA-LYASE*, *CINNAMATE 4-HYDROXYLASE*, *4-COUMAROYL: COENZYME A LIGASE, CHALCONE SYNTHASE*, *CHALCONE ISOMERASE*, *FLAVANONE 3-HYDROXYLASE* (*F3H*) and *FLAVONOID 3′-HYDROXYLASE* (*F3′H*). It is worth mentioning that the first three enzymes provide precursors for all phenylpropanoids, including flavonoids such as flavonols, anthocyanins and proanthocyanidins (Fig. 1). Proanthocyanidin formation is dependent upon the expression of late biosynthesis genes, *DIHYDROFLAVONOL 4-REDUCTASE* (*DFR*), *LEUCOANTHOCYANIDIN REDUCTASE* (*LAR*), *ANTHOCYANIDIN SYNTHASE* ( *ANS* ) and *ANTHOCYANIDIN REDUCTASE* (*ANR*) (Fig. 1). The conversion of flavanones to flavan-3-ols begins with the DFR-mediated stereospecific reduction of dihydroflavonols to leucoanthocyanidins and their subsequent reduction by LAR (Fig. 1; [19, 25–27]). Leucoanthocyanidins are converted to anthocyanidins by ANS [28]. Thereafter, anthocyanidins can be reduced to flavan-3-ols, such as epicatechin in the presence of ANR [18, 29, 30]. In Arabidopsis and *M. truncatula*, proanthocyanidin accumulation and seed coat darkening are also dependent upon the presence of multidrug and toxic extrusion (MATE) transporters, which are involved in ATP-dependent transport of epicatechin 3′-*O*-β-glucoside across the vacuolar tonoplast [31, 32]. These late proanthocyanidin biosynthesis genes are positively regulated by a myeloblastosis proto-oncogene (MYB)-basic helix-loop-helix (bHLH)-WD40 repeat transcription factor complex in seeds, leaves, flowers and fruits; in addition, MYBs are distinguished on the basis of whether their activity activates or represses transcription of proanthocyanidin biosynthesis genes [22, 33–37]. It is postulated that free flavan-3-ols are condensed into proanthocyanidin oligomers/polymers by hitherto unknown enzymes, which are subsequently oxidized [23].

The genome of a *P. vulgaris* Andean landrace, G19833, was recently sequenced, and its annotation was facilitated by RNA-sequencing (RNA-seq) data [3]. RNA-seq overcomes the limitations encountered in traditional transcriptome approaches (e.g., microarrays) as it is capable of detecting low-abundance transcripts [38]. Moreover, the availability of this newly released genome enabled the identification of tissue-specific transcript abundance patterns in developing dry bean plants, as well as those challenged by a fungal pathogen [39, 40]. Recently, research by our group determined that proanthocyanidin B dimers and a C-type trimer, as well as their precursors, catechin and epicatechin, are present at high concentrations in the seed coats of fully mature cranberry beans with known susceptibility to postharvest darkening [5, 41]. By contrast the levels of these metabolites are very low in non-darkening seeds. Together, these metabolite profiles suggest the proanthocyanidin pathway is functional in seed coats of darkening cranberry bean seeds and absent in non-darkening seeds (Fig. 1). In the present study, RNA-seq analysis was used to monitor global transcript abundance profiles in seed coats of darkening and non-darkening cranberry bean recombinant inbred lines (RILs) at three developmental stages in order to test the hypothesis that the accumulation of proanthocyanidins in seed coats of postharvest-darkening susceptible cranberry beans is associated with increased expression of proanthocyanidin metabolism genes.

## Results

### Morphological and proanthocyanidin phenotypes in the seed coats of cranberry bean RILs

**Fig. 2:**
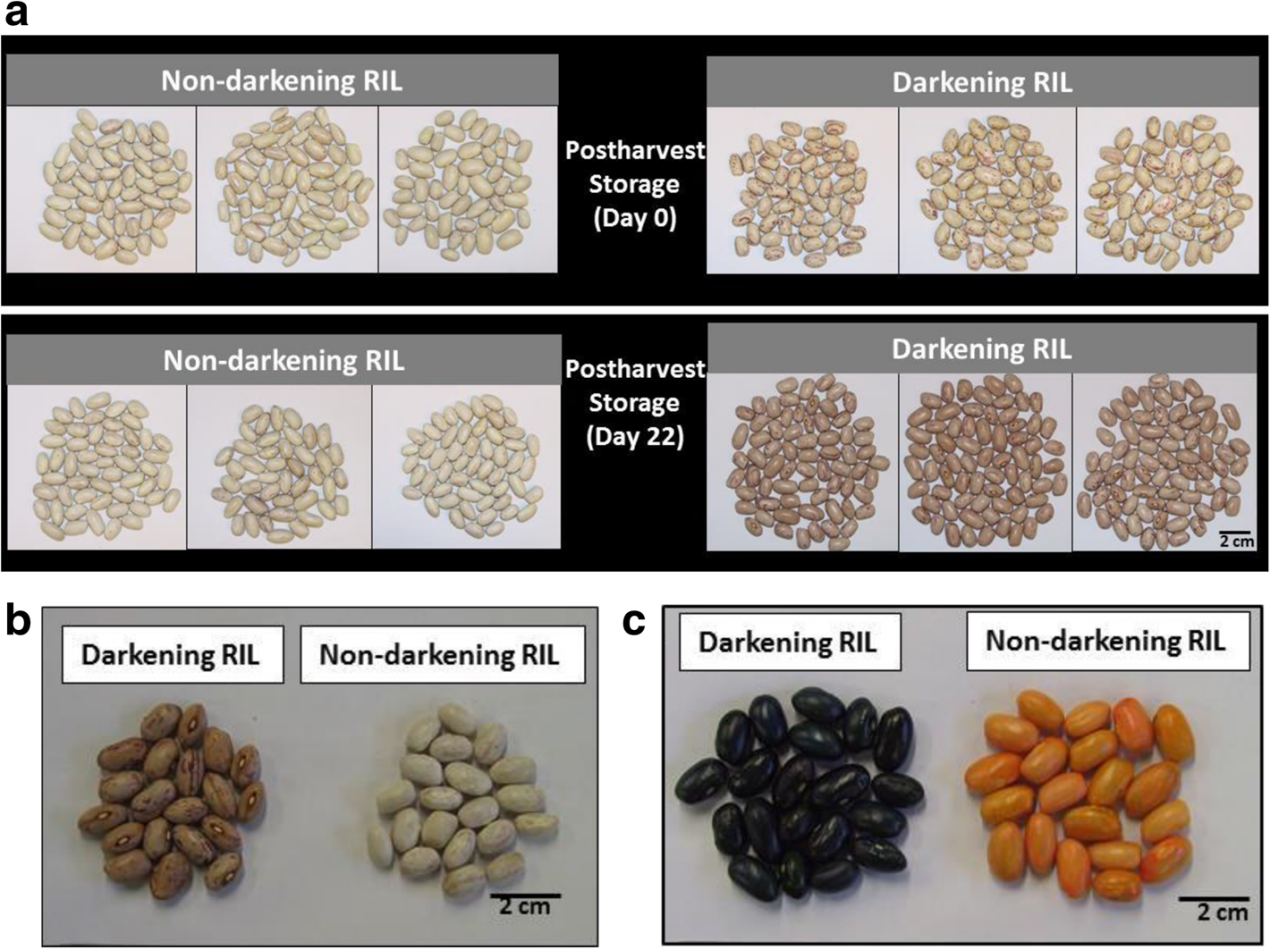
Effect of postharvest storage on seed coat quality of darkening and non-darkening cranberry bean RILs. For both RILs, mature cranberry beans were maintained under (**a**) greenhouse conditions for 22 d or (**b**) in a sealed plastic bag at 4 °C for 48 months (**c**) DMACA staining of aged seeds from both RILs as described under Methods. Scale bar represents 2 cm

RILs were generated from a cross between the postharvest darkening-susceptible cranberry bean ‘Etna’ and the non-darkening cranberry-like bean, ‘Wit-rood boontje’, and herein are referred to as darkening and non-darkening RILs. A qualitative analysis confirmed that a darkening of the seed coat background occurred in beans collected from mature pods of the darkening RIL following storage under greenhouse conditions for 22 days (Fig. 2a). During the same period, there was no change in the seed coat colour background of mature beans sampled from non-darkening RIL plants. Similarly, these visual phenotypes were apparent in seeds left at 4 °C for 48 months (Fig. 2b). These aged seeds were incubated with 4-dimethylaminocinnamaldehyde (DMACA), which interacts with proanthocyanidin terminal units and/or their monomeric precursors in plant tissues [42]. Thereafter, staining was evident in seeds of the darkening RIL, indicating the presence of proanthocyanidins and their related metabolites (Fig. 2c). No staining was evident in aged seeds of the non-darkening RIL.

**Fig. 3:**
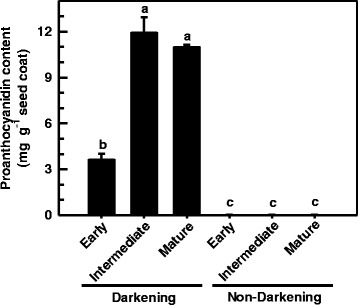
Seed coat proanthocyanidin levels in developing cranberry beans. Total extractable proanthocyanidin levels were determined in seed coats isolated from darkening and non-darkening cranberry bean RILs at early, intermediate and mature stages of bean seed development. Total extractable proanthocyanidin levels are expressed as procyanidin A2 equivalents as described under Methods. Each datum represents the mean ± standard error of three greenhouse replicates. The proanthocyanidin level data were analyzed for statistical differences with a one-way analysis of variance; for both RILs and their developmental stages, means were compared with the Tukey’s test. Shared letters indicate no significant differences at *p* ≤ 0.05

Previously, we determined that high levels of proanthocyanidins and their precursors are present in mature bean seed coats of the darkening RIL, but otherwise absent in the non-darkening RIL seed coats [5]. The aforementioned study did not analyze proanthocyanidin content in seed coats of immature beans. Here, the levels of total extractable proanthocyanidins were measured in the seed coat of both cranberry bean RILs as a function of seed development. This assessment was based on a simple spectrophotometric assay following the incubation of seed coat extracts with acidified DMACA to yield a chromophore having a maximum absorbance at 640 nm [43, 44]. Total extractable proanthocyanidin levels in cranberry bean seed coats were quantified by comparison to a known range of authentic procyanidin A2 dimer standard [41]. Flavan-3-ol standards were not chosen for this comparison as there is a precedent for underestimating proanthocyanidin concentrations [44]. In the darkening cranberry bean RIL, the levels of these metabolites in seed coats of intermediate stage seeds were approximately 2-fold that of the early stage seed coats (Fig. 3). The levels of these metabolites remained unchanged thereafter. By contrast, total extractable proanthocyanidin levels were negligible in seed coats of non-darkening cranberry bean RIL, regardless of seed developmental stage.

### Analysis of the seed coat transcriptome

RNA-seq analysis was used to evaluate whether changes in the seed coat transcriptome were associated with proanthocyanidin levels as a function of seed development in cranberry beans. For maximal read depth, all cDNA libraries were prepared following rRNA depletion, as it is known that this highly abundant RNA strongly interferes with many RNA-seq platforms [45, 46]. The Illumina HiSeq 2500 platform was used to generate paired-end reads for 18 seed coat cDNA libraries, representing three greenhouse replicates of both cranberry bean RILs at early, intermediate and mature stages of seed development. For libraries of both RILs at all three developmental stages, the average number of raw sequence reads of 101 bp length ranged from 50.6 to 57 million (Table 1). The quality trimming procedure generated a total of 889.8 M reads for all 18 seed coat libraries. Bowtie2 and TopHat mapped approximately 95% of these reads to the *P. vulgaris* G19833 reference genome (Version 1.0) [3]. The analysis revealed that 1.5% of the total mapped reads aligned to more than one location in the reference genome. Cufflinks was used to estimate the abundance of ambiguous reads in each biological replicate, including splice variants [47], and this approach yielded an average of 41,746 transcripts across all biological replicates. The original estimation of protein coding loci in *P. vulgaris* was 27,197 [3], whereas 31,638 genes are projected in the *Phaseolus vulgaris* Gene Expression Atlas [39]. Gene counts for all seed coat libraries were normalized in Cuffnorm, yielding an average of 27,751 genes. Transcript levels (expressed as fragments per kilobase of exon per million fragments mapped, FPKM) are provided for all 18 seed coat libraries, including those genes annotated to the *P. vulgaris* genome (see Additional file 1).

**Table 1:** Metrics for cranberry bean seed coat transcripts generated by Illumina sequencing

	Darkening RIL seed coats			Non-Darkening RIL seed coats		
	Early	Intermediate	Mature	Early	Intermediate	Mature
Raw reads	55,936,639 (±5.9%)	50,956,017 (±4.4%)	50,662,455 (± 5.4%)	54,666,751 (± 7.5%)	52,473,537 (±6.2%)	54,424,625 (± 6.4%)
Trimmed reads	51,875,329 (±5.0%)	47,244,783 (± 4.4%)	47,241,601 (± 4.9%)	50,916,550 (± 7.5%)	48,876,790 (± 5.9%)	50,446,588 (± 5.4%)
Total mappings	50,185,242 (± 4.8%)	45,620,314 (± 4.5%)	45,831,924 (± 4.9%)	49,381,726 (± 7.5%)	47,212,581 (± 5.9%)	48,831,478 (± 5.2%)
Single mappings	49,391,395 (± 4.8%)	45,003,334 (± 4.5%)	45,174,334 (±4.8%)	48,592,490 (±7.5%)	46,573,926 (± 5.9%)	48,173,184 (± 5.2%)
Multi mappings	793,847 (± 4.1%)	616,980 (± 6.4%)	657,590 (± 7.7%)	789,237 (± 6.4%)	638,656 (±5.7%)	658,294 (± 5.8%)
Alignment (%)	95.3 (± 0.3%)	95.3 (± 0.2%)	95.7 (± 0.1%)	95.5 (±0.2%)	95.4 (± 0.2%)	95.6 (± 0.2%)
Transcripts	42,715 (±0.7%)	41,368 (± 0.3%)	41,077 (± 0.4%)	42,921 (±0.5%)	41,400 (± 0.2%)	40,993 (± 0.2%)
Gene counts	27,915	27,727	27,661	27,897	27,683	27,626

For each RIL developmental stage, data represents the mean ± percent standard error (denoted in brackets) of three greenhouse replicates.

### Differential gene expression analysis

A total of 1336 genes were differentially expressed between darkening and non-darkening seed coats with a relative expression ratio of ≥1.4, a *P* value ≤0.01 and non-zero raw read counts for one or more cDNA libraries. Moreover, a comparison of developmental stage-specific cDNA libraries revealed genes were differentially expressed between the RILs at early, intermediate and mature stages of seed coat development (Table 2). The differentially expressed genes for each developmental stage were classified into two groups: genes up-regulated in darkening RIL seed coats and genes up-regulated in non-darkening RIL seed coats (see Additional files 2 and 3). Of these, the largest number of differentially expressed genes was apparent at the mature stage of development, with 64% of these upregulated in the darkening RIL seed coats, and the remainder were upregulated in the non-darkening RIL (Table 2). It is worth mentioning that 57 genes were upregulated in darkening RIL seed coats, regardless of developmental stage (see Additional file 2). By contrast, 26 genes were upregulated in the non-darkening RIL seed coats in all three stages analyzed (see Additional file 3). In addition, in both RILs there was evidence for genes upregulated in two of the three stages analyzed (see Additional files 2 and 3). For example, 99 genes were upregulated in seed coats at both early and intermediate stages in the darkening RIL relative to the non-darkening RIL, but unaffected at the mature stage. We determined that 29 genes were differentially expressed in a stage-specific manner in both darkening and non-darkening RILs (e.g., upregulated in early and intermediate stages of the darkening- and non-darkening RIL, respectively; see Additional file 4). The remainder and bulk of the differentially expressed genes were upregulated solely at one developmental stage.

**Table 2:** Summary of differentially expressed genes between darkening and non-darkening RIL seed coats at each developmental stage

Seed developmental stage	Differentially expressed genes (*P* ≤ 0.01)	Up-regulated genes	
		Darkening RIL	Non-darkening RIL
Early	595	361	234
Intermediate	439	262	177
Mature	711	456	255

**Fig. 4:**
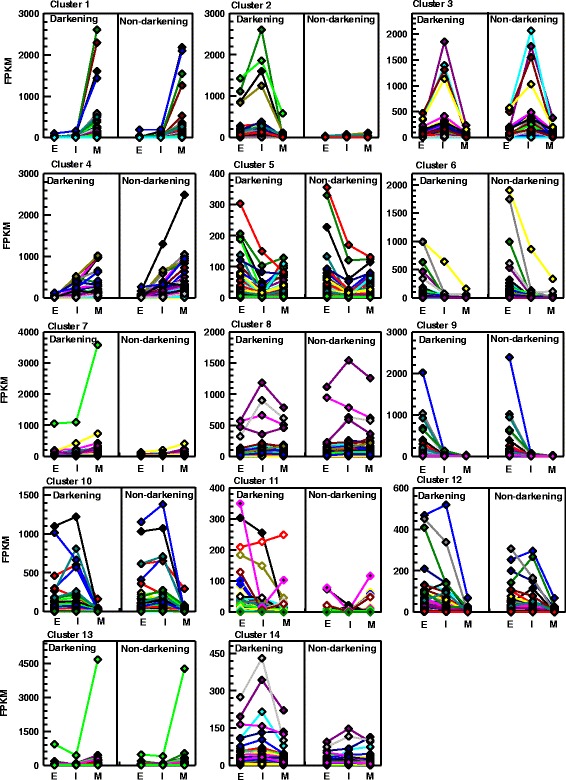
Clustering of differentially expressed seed coat genes in darkening and non-darkening cranberry bean RILs. For the 14 gene clusters, expression patterns demonstrate seed development and/or RIL-specific expression patterns. For each cluster, the transcript levels for individual genes (represented by various coloured lines) are given in FPKM at early (E), intermediate (I) and mature (M) stages of development for the darkening and non-darkening RILs

**Fig. 5:**
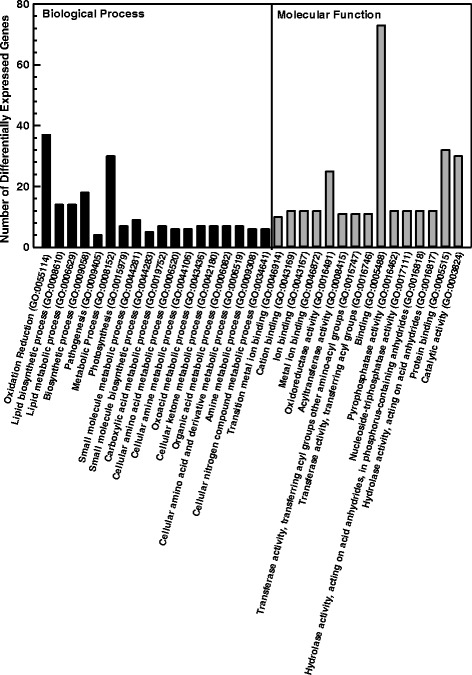
GO Enrichment analysis of differentially expressed genes subjected to a clustering analysis

Using model clustering techniques, the differentially expressed seed coat genes were clustered into 14 groups; the number of genes per cluster ranged from 49 to 168 (Additional file 5). For all clustered genes, their expression patterns across seed maturation stages were visualized after normalization of the raw read counts to FPKM (Fig. 4; see Additional file 5). Genes belonging to cluster 5, 6 and 9 displayed the highest transcript abundance at the early stage, whereas transcript levels were greatest at the intermediate stage in clusters 2, 3, 8 and 14. Moreover, cluster 2 genes were more highly expressed in the darkening RIL relative to the dramatically lower transcript levels in the non-darkening RIL. A similar expression profile pattern was apparent for various genes from cluster 14. Transcript levels were maximal at the mature stage for genes belonging to clusters 1, 4, 7 and 13. Gene ontology (GO) enrichment analysis revealed that 197 differentially expressed genes belonging to clusters 1, 2, 9 and 14 were associated with biological processes, which included metabolic processes related to amino acids, amines, lipids, organic acids, redox processes and small molecules (Fig. 5). In addition, the GO enrichment analysis identified 287 genes belonging to cluster 1, 2, 4, 7 and 9 that were categorized as 15 separate molecular function GO terms, ranging from catalytic activity, hydrolase activity, and metal ion binding. No significant GO terms were associated with genes belonging to cluster 5, 6, 8, and 11–13.

**Fig. 6:**
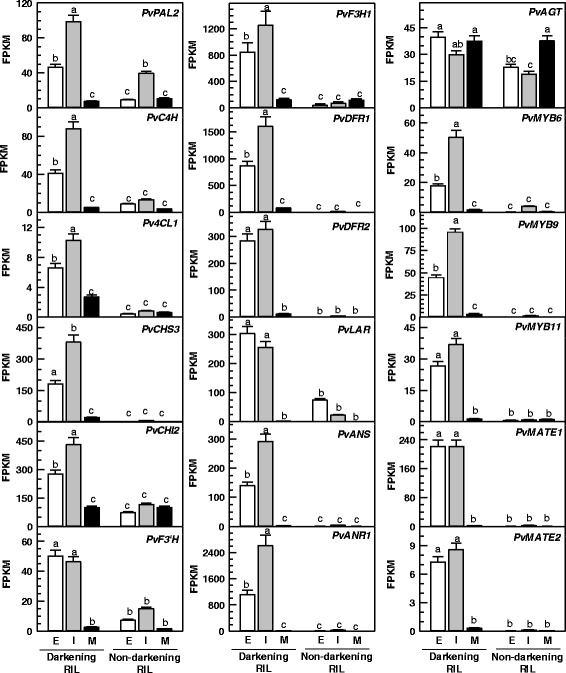
Proanthocyanidin pathway gene expression in seed coats of darkening and non-darkening cranberry beans. Transcript levels for each gene are shown for seed coats isolated from seeds of both RILs at early (E), intermediate (I) and mature (M) stages of development, and are expressed as FPKM. Each datum represents the mean ± standard error of three greenhouse replicates. Transcript level data for each gene were analyzed for statistical differences with a one-way analysis of variance; for both RILs and their developmental stages, means were compared with the Tukey’s test. Shared letters indicate no significant differences at *p* ≤ .05. For each gene transcript, the corresponding gene accession number is provided in brackets: *PvPAL2*, *PHENYLALANINE AMMONIA LYASE 2* (*Phvul.001G177700*, *Phvul.001G177800*); *PvC4H*, *CINNAMATE 4-HYDROXYLASE* (*Phvul.008G247400*); *Pv4CL1*, *4-COUMAROYL:COENZYME A LIGASE 1* (*Phvul.002G040100*); *PvCHS3*, *CHALCONE SYNTHASE 3* (*Phvul.002G038700*); *PvCHI2*, *CHALCONE ISOMERASE 2* (*Phvul.009G143100*); *PvF3′H*, *FLAVONOID 3′-HYDROXYLASE* (*Phvul.009G192400*); *PvF3H1*, *FLAVANONE 3-HYDROXYLASE 1* (*Phvul.003G261900*); *PvDFR1*, *DIHYDROFLAVONOL 4-REDUCTASE 1* (*Phvul.001G012700*); *PvDFR2*, *DIHYDROFLAVONOL 4-REDUCTASE 2* (*Phvul.001G012800*); *PvLAR*, *LEUCOANTHOCYANIDIN REDUCTASE* (*Phvul.007G102100*); *PvANS*, *ANTHOCYANIDIN SYNTHASE* (*Phvul.002G152700*); *PvANR1*, *ANTHOCYANIDIN REDUCTASE 1* (*Phvul.002G218700*); *PvAGT*, *URIDINE DIPHOSPHATE-GLUCOSE: ANTHOCYANIDIN 3-O-GLUCOSYLTRANSFERASE* (*Phvul.002G214300*); *PvMYB6*, *MYELOBLASTOSIS PROTO-ONCOGENE 6* (*Phvul.006G114800*); *PvMYB9*, *MYELOBLASTOSIS PROTO-ONCOGENE 9* (*Phvul.011G105600*); *PvMYB11*, *MYELOBLASTOSIS PROTO-ONCOGENE 11* (*Phvul.003G222400*); *PvMATE1*, *MULTIDRUG AND TOXIN EXTRUSION 1* (*Phvul.008G197000*); *PvMATE2*, *MULTIDRUG AND TOXIN EXTRUSION 2* (*Phvul.006G028700*)

The GO enrichment analysis identified several genes belonging to cluster 2 as biosynthetic genes (see Additional file 5). Upon further examination, it was determined that many of these genes were annotated as flavonoid/proanthocyanidin biosynthesis genes in the *P. vulgaris* genome. Furthermore, these were classified here on the basis of their similarity at the amino acid level to known structural and regulatory proanthocyanidin pathway genes from other plants, such as Arabidopsis, *M. truncatula*, *Glycine max* and *Vitis* species. Thus we identified changes in their respective seed coat transcript levels as a function of seed development (Fig. 6). The late proanthocyanidin biosynthesis genes, *PvF3H1*, *PvDFR1*, *PvLAR*, *PvANS* and *PvANR1* were expressed at all stages of seed maturation in the darkening cranberry bean RIL. The highest transcript levels were detected in cDNA libraries prepared from seed coats of intermediate stage beans. Thereafter, a decline in transcript abundance was apparent at the mature stage for all three genes. Conversely, transcript levels were very low in seed coats of the non-darkening RIL, regardless of developmental stage. A second DFR gene, *PvDFR2*, was also identified, but transcript levels were not different in early and intermediate stage seed coats. In addition, *PvDFR2* transcript levels in darkening beans were 15 to 32% lower than those apparent for *PvDFR1*. Similar transcript profile patterns were apparent for the late biosynthesis gene *PvANS*. For early biosynthesis genes (e.g., *PHENYLALANINE AMMONIA-LYASE 2*), transcript abundance levels in the darkening RIL were comparatively lower than the aforementioned genes, although for the most part expression peaked at the intermediate stage. A number of genes were annotated as proanthocyanidin pathway transporters (e.g., *PvMATE1*) and transcription factors (e.g., *PvMYB6* and *PvMYB11*). In general, their respective transcript levels peaked at the intermediate stage and were as much as two orders of magnitude lower than that of the late biosynthesis structural genes in the darkening RIL, although 1.4 to 410 times greater than the non-darkening RIL. Genes annotated as *bHLH* and *WD40 REPEAT* transcription factors were also upregulated in the darkening RIL, although none of the *bHLH* candidates belonged to cluster 2 (see Additional files 2 and 5).

**Fig. 7:**
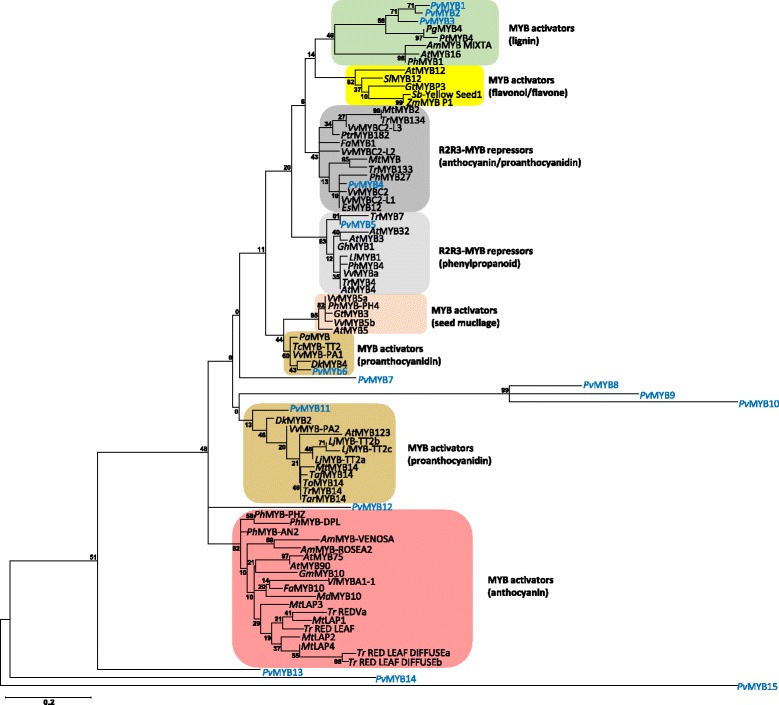
Phylogenetic comparison of *P. vulgaris* MYB amino acid sequences with known repressor and activator MYBs from other plant species. In silico translations of *P. vulgaris* MYB coding sequences corresponding to genes that were differentially expressed between darkening and non-darkening cranberry bean RILs were aligned to amino acid sequences of other plant MYBs using ClustalW (www.genome.jp/tools/clustalw; [81]). The maximum likelihood method in MEGA 6.06 was used to construct the unrooted tree [82]. Numbers proximal to each node represent the percent support values from the bootstrap analysis using 500 iterations. Previously characterized MYBs are represented in black font, and *Pv*MYBs are represented in blue font. GenBank™ accession numbers for each MYB are provided in parentheses: *Antirrhinum majus* MYB MIXTA, *Am*MYB MIXTA (CAA55725.1); *Am*MYB-ROSEA2 (ABB83827.1); *Am*MYB-VENOSA (ABB83828.1); *Arabidopsis thaliana* MYB3, *At*MYB3 (AAS10027.1); *At*MYB4 (AAP13410.1); *At*MYB5 (AEE75369.1); *At*MYB12 (AEC10843.1); *At*MYB16 (ABI49476.1); *At*MYB32 (ABD91498.1); *At*MYB75 (AAG09100.1); *At*MYB90 (AEE34503.1); *At*MYB123 (AAK54744.1); *Diospyros kaki* MYB2, *Dk*MYB2 (BAI49719.1); *Dk*MYB4 (BAI49721.1); *Epimedium sagittatum* MYB12, *Es*MYB12 (AFH03064.1); *Fragaria* x *ananassa* MYB1, *Fa*MYB1 (AAK84064.1); *Fa*MYB10 (ABX79947.1); *Gossypium hirsutum* MYB1, *Gh*MYB1(AAN28270.1); *Garcinia mangostana* MYB10, *Gm*MYB10 (ACM62751.1); *Gentiana triflora* MYB, *Gt*MYB3 (BAF96933.1); *Gt*MYBP3 (BAM71801.1); *Leucaena leucocephala* MYB1, *Ll*MYB1(ADY38393.2); *Lotus japonicus* MYB TT2a, *Lj*MYB-TT2a (BAG12893.1); *Lj*MYB-TT2b (BAG12894.1); *Lj*MYB-TT2c (BAG12895.1); *Malus* x *domestica* MYB10, *Md*MYB10 (ACQ45201.1); *Medicago truncatula* MYB, *Mt*MYB (KEH30894.1); *Mt*MYB2 (AES99346.1); *Mt*MYB14 (AFJ53057.1); *Mt*LAP1 (ACN79541.1); *Mt*LAP2 (ACN79539.1); *Mt*LAP3 (ACN79542.1); *Mt*LAP4 (ACN79540.1); *Prunus avium* MYB, *Pa*MYB (ADY15314.1); *Picea glauca* MYB4, *Pg*MYB4 (ABQ51220.1); *Petunia* x *hybrida* AN2, *Ph*MYB-AN2 (AAF66727.1); *Ph*MYB1 (CAA78386.1); *Ph*MYB4 (ADX33331.1); *Ph*MYB PH4 (AAY51377.1); *Ph*MYB-DPL (ADW94950.1); *Ph*MYB27 (AHX24372.1); *Ph*MYB-PHZ (ADW94951.1) *Pinus taeda* MYB4, *Pt*MYB4 (AAQ62540.1); *Populus tremula* x *Populus tremuloides*, *Ptr*MYB182 (AJI76863.1); *Solanum lycopersicum* MYB12*, Sl*MYB12 (ACB46530.1); *Sorghum bicolor* Yellow Seed1, *Sb*-Yellow Seed1 (AAX44239.1); *Theobroma cacao* Transparent Testa 2-Like MYB, *Tc*MYB-TT2 (ADD51352.1); *Trifolium affine* MYB14, *Ta*MYB14 (AFJ53046.1); *Trifolium arvense* MYB14, *Tar*MYB14 (AFJ53053.1); *Trifolium occidentale* MYB14, *To*MYB14 (AFJ53052.1); *Trifolium repens* MYB4, *Tr*MYB4 (AMB27079.1); *Tr*MYB7 (AMB27080.1); *Tr*MYB14 (AFJ53050.1); *Tr*MYB133 (AMB27081.1); *Tr*MYB134 (AMB27082.1); *Tr* RED LEAF (AIT76557.1); *Tr* RED LEAF DIFFUSEa (AIT76556.1); *Tr* RED LEAF DIFFUSEb (AIT76560.1); *Tr* REDVa (AIT76565.1); *Vitis labrusca* x *Vitis vinifera* MYBA1–1, *Vl*MYBA1–1 (BAC07537.1); *Vitis vinifera* MYBPA1, *Vv*MYBPA1 (CAJ90831.1); *Vv*MYB4a (ABL61515.1); *Vv*MYB5a (AAS68190.1); *Vv*MYB5b (AAX51291.1); *Vv*MYBC2 (ABW34393.1); *Vv*MYBC2-L1 (AFX64995.1); *Vv*MYBC2-L2 (ACX50288.1); *Vv*MYBC2-L3 (AIP98385.1); *Vv*MYB-PA2 (ACK56131.1); *Zea mays* MYB P1, *Zm*MYB P1 (ABM21535.1). In silico translations of cranberry bean MYBs were annotated as *Pv*MYB1 to *Pv*MYB15 and their corresponding gene accession numbers are provided in parentheses: *Pv*MYB1 (*Phvul.001G219000*); *Pv*MYB2 (*Phvul.001G221500*); *Pv*MYB3 (*Phvul.003G203900*); *Pv*MYB4 (*Phvul.002G092100*); *Pv*MYB5 (*Phvul.009G158200*); *Pv*MYB6 (*Phvul.006G114800*); *Pv*MYB7 (*Phvul.009G228200*); *Pv*MYB8 (*Phvul.005G114100*); *Pv*MYB9 (*Phvul.011G105600*); *Pv*MYB10 (Phvul.002G163500); *Pv*MYB11 (*Phvul.003G222400*); *Pv*MYB12 (*Phvul.002G306000*); *Pv*MYB13 (*Phvul.004G053600*); *Pv*MYB14 (*Phvul.007G093100*); *Pv*MYB15 (*Phvul.001G227900*). Scale bar represents 0.2 amino acid substitutions per site

A comparison of in silico translations of all differentially expressed *PvMYB*s with amino acid sequences of known MYBs from other plant species revealed *Pv*MYB6 and *Pv*MYB11 were phylogenetically similar to MYBs from other plant species that are known to positively regulate proanthocyanidin biosynthesis gene expression (Fig. 7). Moreover, *Pv*MYB6 and *Pv*MYB11 were well separated from clades containing R2R3-MYBs that negatively regulate proanthocyanidin/anthocyanin biosynthesis in various plant species. None of the differentially expressed *Pv*MYBs clustered with MYBs known to activate the biosynthesis of flavone/flavonol, or with those involved in anthocyanin biosynthesis and seed mucilage production. For all differentially expressed genes identified in this study, a transcription factor binding site (TFBS) enrichment analysis was performed to determine whether sequences upstream of the transcription start site (−500 to −1 bp) contained putative MYB and bHLH binding sites similar to those known for Arabidopsis and *Brassica napus* proanthocyanidin biosynthesis genes [20, 48, 49]. The TFBS enrichment analysis revealed the percentage of genes containing MYB and bHLH binding sites within regions upstream of the transcription start site were comparable for genes upregulated in the darkening RIL versus the non-darkening RIL (Table 3). By contrast, the TFBS analysis revealed a higher percentage of transcription factor binding sites were present in the regions upstream of cluster 2 genes relative to the complete list of genes upregulated in darkening cranberry beans.

**Table 3:** Analysis of the conserved MYB and bHLH core consensus sequences in the region upstream of the putative transcription start site of differentially expressed seed coat genes

	Conserved regulatory sequence	Percentage of genes containing putative binding site		
		Non-darkening RIL upregulated genes	Darkening RIL upregulated genes	Cluster 2 genes
MYB-core	C[AGCT]GTT[AG]	53.8	55.6	72.5
bHLH E-box	CACGTG	19.7	26.1	66.7

The percentage of genes containing a putative binding site is expressed as the number of genes containing the conserved regulatory sequence divided by the total number of genes in the group. The total number of annotated genes in each group is provided in brackets: Non-darkening RIL upregulated genes (529); Darkening RIL upregulated genes (804); Cluster 2 genes (51)

**Fig. 8:**
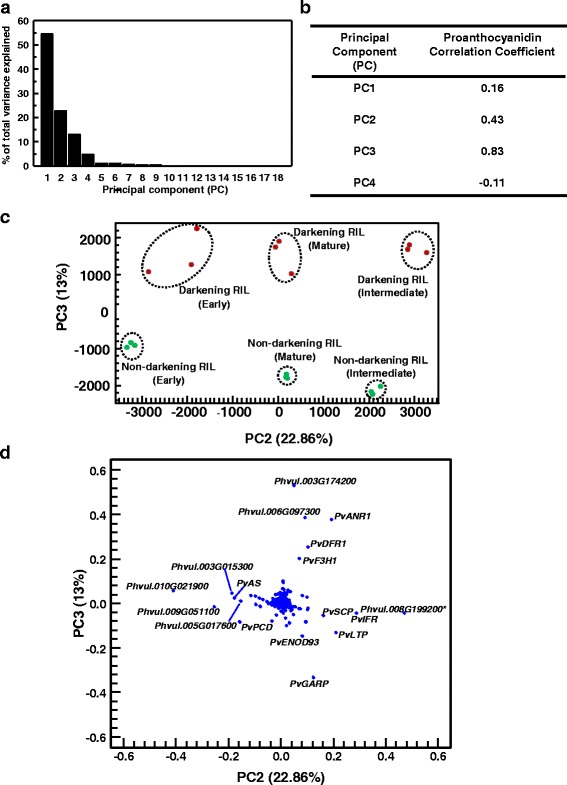
Relationship between proanthocyanidin levels and gene expression in cranberry bean seed coats. **a** Scree plot of the percentage of total variance explained by each principal component (PC). **b** Correlation matrix of the relationship between seed coat total proanthocyanidin levels and PCs accounting for 95% of the total variance. For each comparison, the strength of the correlation is represented by the corresponding correlation coefficient value. **c** Score plot of PC2 versus PC3. Biological replicates of darkening and non-darkening RIL seed coat are represented by *red* and *green* symbols, respectively. Dashed circles within the score plot are used to identify grouping of darkening and non-darkening RIL seed coat transcriptomes at each developmental stage. **d** Correlation loading plot analysis of PC2 versus PC3. Scatter dots represent the contribution of each of the 1336 differentially expressed genes. Genes with absolute coefficients greater than 0.1 for PC2 and/or PC3 were labelled with their corresponding gene annotation or accession numbers. *Pv*, *Phaseolus vulgaris*; *PvANR1*, *ANTHOCYANIDIN REDUCTASE 1* (*Phvul.002G218700*); *PvAS*, *ASPARAGINE SYNTHETASE* (*Phvul.006G069300*); *PvDFR1*, *DIHYDROFLAVONOL 4-REDUCTASE 1* (*Phvul.001G012700*); *PvENOD93*, *EARLY NODULIN 93* (*Phvul.003G162200*); *PvF3H1*, *FLAVANONE 3-HYDROXYLASE 1* (*Phvul.003G261900*); *PvGARP*, *GIBBERELLIC ACID REGULATED PROTEIN* (*Phvul.001G006300*); *PvIFR*, *ISOFLAVONE REDUCTASE* (*Phvul.009G059000*); *PvLTP*, *LIPID TRANSFER PROTEIN* (*Phvul.008G137100*); *PvPCD*, *PROGRAMMED CELL DEATH PROTEIN* (*Phvul.004G174800*); *PvSCP*, *SERINE CARBOXYPEPTIDASE* (*Phvul.011G010600*)

In order to assess which of the aforementioned proanthocyanidin pathway genes were most highly associated with proanthocyanidin accumulation in seed coats of the darkening RIL, a principal component analysis (PCA) was performed for the transcript abundance profiles of all 1336 differentially expressed genes. To this end, the normalized gene expression data (represented as FPKM) for all 18 RNA-seq libraries were converted into 18 uncorrelated variables, herein referred to as principal components (PCs). PCs 1 to 4 accounted for 95.2% of total variance (Fig. 8a). In order to determine which of these PCs accounted for proanthocyanidin accumulation within the seed coats of the darkening cranberry bean RIL, a correlation analysis was performed. Positive correlation coefficients were observed between total proanthocyanidin levels and PCs 1, 2 and 3 (Fig. 8b), with the largest influence attributable to PC3. The score plots revealed that PC2 explained 22.86% of the total variance, yielding a clear separation of transcript profiles for all three developmental stages. In addition, PC3 explained 13% of the total variance, and transcript profiles for the darkening RIL were separated from those of the non-darkening RIL (Fig. 8c). To identify which gene transcript levels were associated with the difference in proanthocyanidin levels between darkening and non-darkening RILs, a correlation loading plot analysis was implemented for all differentially expressed genes (Fig. 8d). Here, five genes displayed high positive coefficients for both PCs, and were associated with proanthocyanidin accumulation. This included transcript profiles for two genes of unknown function, *Phvul.006G097300* and *Phvul.003G174200*. In silico translation revealed these encode small proteins of 66 and 73 amino acids, respectively. In the darkening RIL, transcripts for the *Phvul.003G174200* gene were greatest at the mature stage of bean development and were 2.3-fold that of the levels apparent at early and intermediate stages (see Figure S1 in Additional file 6). By comparison, *Phvul.*006G097300 transcript levels were decreased at the mature stage relative to the immature developmental stages in the darkening RIL. In either case, expression of these unknown genes was minimal in seed coats of the non-darkening RIL. Clustering analyses can identify groups of genes with similar expression patterns; moreover, this information can be used to infer the biological function of unknown genes based on their association with genes of known function [50]. *Phvul.006G097300* belonged to cluster 2 genes, many of which are annotated as flavonoid/proanthocyanidin structural and regulatory genes (see Additional file 5). A BLAST search of the non-redundant protein database in NCBI determined that the in silico translation of *Phvul.006G097300* has similarity to small proteins of hypothetical function, including an adzuki bean leucine-rich repeat extensin-like protein. *Phvul.003G174200* belonged to cluster 7, which was comprised of many genes involved in DNA binding. Interestingly a GO enrichment analysis revealed that genes encoding binding proteins (GO:0005488) represented the largest group of differentially expressed seed coat genes (Fig. 6). Apart from these unknown genes, proanthocyanidin accumulation was strongly associated with an increase in transcripts corresponding to the proanthocyanidin biosynthesis genes *PvF3H1*, *PvDFR1*, and *PvANR1*. *PvANR1* transcript levels were more strongly associated with proanthocyanidin levels in the darkening RIL cranberry bean than *PvF3H1* and *PvDFR1* transcript levels (Fig. 8).

### Biochemical properties of a recombinant cranberry bean ANR

**Fig. 9:**
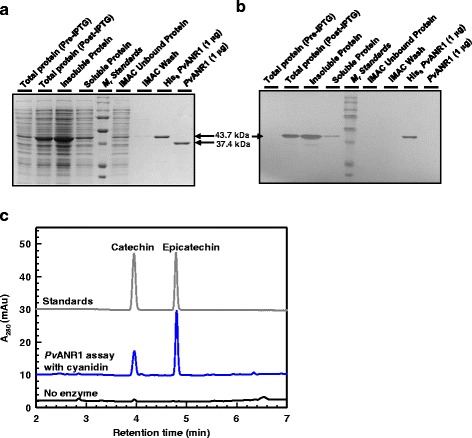
Biochemical analysis of the recombinant *Pv*ANR1 enzyme. Coomassie Brilliant Blue stained SDS-PAGE (**a**) and immunoblot (**b**) analysis of the expression and purification of the recombinant *Pv*ANR1 from *E. coli* BL21 cells. With exception of lanes where corresponding protein amount is indicated, lanes were loaded by equal volume (15 μL) in a final of 1× SDS sample buffer. The immunoblot was subjected to a chromogenic stain following successive probing with an anti-His_6_ antibody and a secondary antibody conjugated to alkaline phosphatase. Abbreviations include: His_6_, hexahistidine; IMAC, immobilized metal affinity chromatography; IPTG, isopropyl β-D-thiogalactopyranoside; *M*_*r*_, relative molecular mass. **c** HPLC-DAD analysis of in vitro *Pv*ANR1 activity. The grey chromatogram represents authentic (−)-epicatechin and (+)-catechin standards. The blue chromatogram represents products formed from assays containing *Pv*ANR1, 100 μM cyanidin and 800 μM NADPH. The black chromatogram represents the analysis of an assay performed in the absence of *Pv*ANR1

As part of this study, it was our aim to investigate the biochemical properties of *Pv*ANR1 due to the strong association between the transcriptional regulation of this putative proanthocyanidin biosynthetic gene and the accumulation of these metabolites in seed coats of the darkening cranberry bean RIL. Recombinant *Pv*ANR1 was expressed and purified from *Escherichia coli*. Denaturing gel electrophoresis and immunoblotting revealed the eluate collected from an immobilized metal affinity chromatography (IMAC) step contained a single hexahistidine (His_6_)-tagged polypeptide of 43.7 kDa (Fig. 9). Immunoblot analysis demonstrated that the subsequent incubation with enterokinase removed the His_6_-tag, yielding a homogenous preparation of a 37.4 kDa polypeptide matching the predicted molecular mass of this protein. For all recombinant protein preparations, approximately 26 ± 2.4% of the His_6_-tag free *Pv*ANR1 was recovered after the enterokinase cleavage step. With this purification strategy, a 6 L bacterial culture yielded an average of 6.75 ± 1.05 mg of recombinant *Pv*ANR1.

A phylogenetic comparison revealed that the *Pv*ANR1 amino acid sequence is closely related to other legume ANRs, including pea and soybean representatives that are expressed in seed coats and utilize cyanidin as a substrate (See Figure S2 in Additional file 6). In vitro *Pv*ANR1 activity was assessed in the presence of cyanidin, the predominant anthocyanidin occurring in seed coats of the darkening cranberry bean RIL [5], and the hydride donor NADPH. When *Pv*ANR1 was incubated with fixed concentrations of NADPH and cyanidin at pH 7.0, HPLC-DAD analysis revealed the formation of two peaks at retention times 3.9 and 4.7 min, which co-migrated with authentic standards of catechin and epicatechin, respectively (Fig. 9c). There was no evidence of spontaneous formation of these products in assays performed in the absence of *Pv*ANR1. The kinetic properties for cyanidin and NADPH were established using this HPLC-DAD based assay (Table 4). For *Pv*ANR1, plots of cyanidin concentration versus the rate of epicatechin and catechin formation did not fit a Michaelis-Menten relationship. A non-linear regression model determined that the *K*_*0.5*_ and apparent *V*_*max*_ for cyanidin-derived epicatechin and catechin formation were highly similar. Interestingly, product formation was dramatically inhibited at cyanidin concentrations greater than the *K*_*0.5*_; the observed *K*_*i*_ for epicatechin and catechin formation were 4.8 and 4.2-fold higher than the *K*_*0.5*_ for these products, respectively. Similarly, a sigmoidal relationship was observed for plots of epicatechin and catechin formation as a function of NADPH concentration in *Pv*ANR1 assays performed at a fixed cyanidin concentration of 100 μM. The highest specificity constant (*K*_*cat*_ */K*_*0.5*_) was revealed for cyanidin-derived epicatechin formation.

**Table 4:** Kinetic parameters for *Pv*ANR1 in assays containing varying amounts of cyanidin and NADPH

Varying substrate	Product	*K*_*0.5*_ (μM)	*K*_*i*_ (μM)	*V*_*max*_ (nmol min^−1^ mg^−1^)	*k*_*cat*_ (s^−1^)	*k*_*cat*_ */K*_*0.5*_ *(*s^−1^ M^−1^)
Cyanidin	Epicatechin^a^	114.2 ± 19.0	631.5 ± 119.9	11.57 ± 1.13	0.0072 ± 0.0007	65.4 ± 13.8
Cyanidin	Catechin^a^	116.5 ± 10.7	583.8 ± 116.1	8.32 ± 0.73	0.0052 ± 0.0004	45.4 ± 6.42
NADPH	Epicatechin^b^	103.5 ± 7.4	-	3.34 ± 0.21	0.0021 ± 0.0001	20.1 ± 0.49
NADPH	Catechin^b^	102.3 ± 7.8	-	2.44 ± 0.18	0.0015 ± 0.0001	14.9 ± 1.03

The *K*_*cat*_ (also referred to as turnover rate) was calculated using a molecular mass of 37.4 kDa for the final recombinant *Pv*ANR1 preparation, following removal of the His_6_ tag

^a^A non-linear regression model for substrate inhibition (as described under Methods) was used to determine apparent kinetic parameters for cyanidin. These assays were performed at a fixed NADPH concentration of 800 μM

^b^The Hill equation was utilized to determine kinetic parameters for NADPH. These assays were performed at a fixed cyanidin concentration of 100 μM; the Hill coefficient for epicatechin and catechin formation was 2.5 ± 0.19 and 2.5 ± 0.15, respectively

### Seed germination

**Fig. 10:**
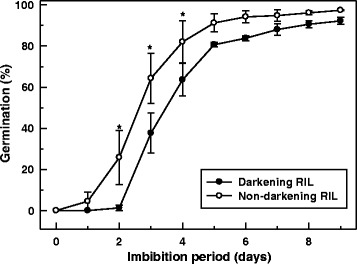
Seed germination rates in darkening and non-darkening cranberry beans. For both RILs, aged mature cranberry beans were sown on sterile agar plates and incubated at 25 °C under darkness for 9 d, as described under Methods. For each RIL in the experiment, the seed germination percentage was determined daily and represents the number of seeds exhibiting radicle emergence relative to the total number of seeds. Each datum represents the mean ± standard error of three separate experiments. The seed germination percentage data were analyzed for statistical differences with a one-way analysis of variance; within each day of the time course, means were compared with the Tukey’s test. *Asterisks* are used to indicate significant differences at *p* ≤ 0.05

In order to assess the impact of seed coat proanthocyanidins and darkening on seed germination, we analysed the percentage of aged seeds exhibiting emerged radicles as a function of imbibition time. On average, 26% of non-darkening seeds germinated after 2 d, whereas no germinated seeds were observed for the beans of the darkening RIL during this period (Fig. 10). Thereafter, an increase in germination percentage was apparent for both RILs, although these proportions were 25 and 20% higher in non-darkening relative to darkening seeds on d 3 and 4, respectively. After 9 d, germination percentages were 92% or higher, and not statistically different between seeds of both RILs.

## Discussion

### Proanthocyanidins accumulated with development in seed coats of a cranberry bean RIL susceptible to postharvest darkening

Plant tissues and their derived foodstuffs are the sole source of proanthocyanidins, including baking chocolate, cinnamon, grape seed, sorghum, chokeberries and dry beans [51]. Moreover, these metabolites exert numerous benefits in humans, including antioxidant and cardioprotective effects [42]. Unfortunately, the presence of these polyphenolic compounds is associated with darkening in dicotyledonous seed coats [15, 18–21]. Seed coat darkening tends to occur in susceptible legumes, such as faba beans, and certain cultivars of edible dry bean, including pinto and cranberry beans [8, 10, 52]. This was also evident in seeds of a cranberry bean darkening RIL derived from a cross between the postharvest darkening susceptible parent ‘Etna’ and the non-darkening ‘Wit-rood boontje’, but otherwise absent in a non-darkening RIL (Fig. 2a, b). Here, we report darkened cranberry beans were DMACA-stained and contained dramatically more total extractable proanthocyanidin levels than its non-darkening counterpart at all stages of seed development (Figs. 2c and 3). For mature stage seed coats, these trends are in agreement with an HPLC-MS analysis of total extractable proanthocyanidin metabolite levels [5]. It is worth mentioning that the current study reports an approximately 100% higher level of seed coat proanthocyanidins in mature darkening RIL beans relative to our earlier study. Quantification in the previous study was based on catechin equivalents; the molecular mass of this compound is 50% that of the procyanidin A2 standard employed in Fig. 3. Moreover, the chromogenic response generated for procyanidin A2 in the in vitro DMACA assay is less than that observed for catechin [44]. Proanthocyanidin levels were greater at the intermediate and mature stages in darkening cranberry bean seed coats. This is not without precedent as proanthocyanidins tend to be largely absent or minimal at early stages of seed development in Arabidopsis and pea, but are increased thereafter [19, 24].

### Proanthocyanidin accumulation in darkening cranberry bean seeds was associated with the co-ordinated upregulation of proanthocyanidin metabolism genes

An RNA-seq approach revealed that 1336 genes were differentially expressed in seed coats of a darkening cranberry bean versus those of a non-darkening genotype. Our findings are consistent with a transcriptome analysis of *Brassica juncea* seed coat genes, which reported 1304 genes are differentially expressed between a brown seed line (proanthocyanidin containing) and a yellow seed line (proanthocyanidin deficient) [53]. Moreover, the majority of the differentially expressed genes in *B. juncea* seed coats are not associated with the proanthocyanidin pathway, which is consistent with the majority of the differentially expressed seed coat genes identified in this study (see Additional files 2, 3 and 5).

A MYB-bHLH-WD40 repeat complex encoded by *TT2*–*TT8*–*TTG1* drives expression of late proanthocyanidin biosynthesis genes (e.g., the ANR gene *BANYULS*) in developing Arabidopsis seeds [24, 33, 35]. In our study, a TFBS analysis of all cluster 2 genes (predominantly flavonoid/proanthocyanidin metabolism genes) revealed an enrichment in putative MYB and bHLH binding sites that match those known for Arabidopsis (Table 3) [20, 48, 49]. In fact, transcript levels for *PvMYB6*, *PvMYB9* and *PvMYB11* were co-ordinately enhanced in darkening cranberry beans and negligible in the non-darkening genotype (Fig. 6). In addition, their expression patterns were correlated with those of proanthocyanidin structural genes (Figs. 4 and 6). Interestingly, *PvMYB6* and *PvMYB11* belong to two separate phylogenetic clades containing MYBs known to activate expression of proanthocyanidin biosynthesis genes (Fig. 7). All of this information taken together indicates that the transcriptional activation of late proanthocyanidin biosynthesis genes is critical for proanthocyanidin content in the seed coats of cranberry beans.

In our study, transcript profiles for three proanthocyanidin biosynthesis genes, *PvF3H1*, *PvDFR1* and *PvANR1* were highly associated with proanthocyanidin accumulation (Fig. 8). Similarly, the expression of ANR genes is restricted to proanthocyanidin accumulating-cells in seed coats of *B. napus* and Arabidopsis [20, 49]. Furthermore, *ANR* expression is well associated with proanthocyanidin accumulation in seed coats of pea and soybean [18, 19]. *PvANR1* transcript levels were negligible in the seed coat of the non-darkening cranberry bean RIL. This finding is consistent with the reduced expression of *ANR* in red-brown soybean seeds, as opposed to the brown seed coat present in cultivars displaying a non-defective *ANR* gene [18]. Interestingly, the *P. vulgaris* genome contains a second *ANR*, annotated here as *PvANR2*, which was phylogenetically similar to a ubiquitously expressed *ANR2* from *Glycine max* [18] (see Figure S2 in Additional file 6). *PvANR2* transcript levels were dramatically lower than *PvANR1*, and not differentially expressed in the RNA-seq analysis investigated in this study. It is worth mentioning that the PCA analysis did not identify *PvLAR* as one of the genes associated with proanthocyanidin accumulation in darkening cranberry beans. This is most likely due to the fact that this gene was expressed at early and intermediate stages in the non-darkening RIL, albeit at lower levels than the darkening RIL (Fig. 6). Similarly, *LAR* is expressed in developing *M. truncatula* seeds, but unlike *DFR*, *ANS* and *ANR*, its transcript profiles are not well associated with proanthocyanidin accumulation [54]. Conversely, LAR and ANR contribute to the respective production of catechin and epicatechin in pea seeds and *Theobroma cacao* [19, 27]. Thus, the possibility remains that LAR contributes to proanthocyanidin biosynthesis in cranberry bean seed coats.

### Recombinant *Pv*ANR1 produced catechin and epicatechin

Seed coats of mature cranberry beans of the darkening RIL contain high levels of catechin and epicatechin, as well as their proanthocyanidin dimers and trimers [5]. Here, we purified a recombinant *Pv*ANR1 following its expression in *E. coli* (Fig. 9a). The molecular mass of the recombinant *Pv*ANR1 (37.4 kDa) is similar to that of *Gm*ANR1 [18]. In vitro biochemical assays revealed that in the presence of the hydride donor NADPH, cyanidin was converted into products that co-chromatographed with authentic catechin and epicatechin standards (Fig. 9c). A kinetic analysis of this enzyme determined that these products were formed with similar catalytic efficiencies (Table 4). For *Pv*ANR1, the apparent *V*_*max*_ for epicatechin formation from cyanidin is within the range of those detected for other ANRs [19, 55]. Similarly, recombinant ANRs from Arabidopsis, *Gossypium hirsutum*, *M. truncatula*, *Vitis bellula* and *Camellia sinensis* form both flavan-3-ol products in vitro [29, 55–58]. Moreover, the intrinsic epimerase activity of a *V. vinifera* ANR promotes the stereospecific reduction of cyanidin at the C2 and C4 positions to form both (+)-epicatechin and (−)-catechin [59]. It is unclear as to whether a similar mechanism is apparent for *Pv*ANR1, as chiral chromatography was not used in our study. Together with the transcriptome analysis, the in vitro biochemistry of the recombinant *Pv*ANR1 suggests it is a major enzyme involved in the production of proanthocyanidin precursors in cranberry bean, although the possibility remains that LAR activity could also contribute to catechin formation in cranberry bean seeds. At concentrations above the apparent *K*_*0.5*_ for cyanidin, *Pv*ANR1 activity was inhibited by this substrate. This is not without precedent as reduced specific activities are evident for *Gm*ANR1 at cyanidin concentrations in excess of 100 μM [18]. Moreover, non-hyperbolic kinetic relationships have been described for a recombinant *V. bellula* ANR enzyme [58]. In terms of the biological significance, this could represent a mechanism for feed-forward inhibition of this enzyme as a means of limiting the over-accumulation of proanthocyanidins, and allowing ample substrate for simultaneous anthocyanin formation in cranberry bean seed coats.

### Seed coat germination was delayed in non-darkening cranberry bean seeds

In darkening cranberry seeds, germination was delayed by 1 d and consistently lower over the first 4 d of imbibition relative to the non-darkening RIL (Fig. 10). This is most likely due to the dramatic difference in proanthocyanidin content within the seed coats of these two genotypes. This is in agreement with a report demonstrating that germination is inhibited in Arabidopsis and *B. napus* seeds following the application of exogenous proanthocyanidins [60]. In Arabidopsis, seed coats that are high in proanthocyanidins promote strong seed dormancy, as these are less permeable to water and promote *de novo* formation of the growth inhibitor, abscisic acid [60, 61]. The accelerated germination capacity in non-darkening cranberry bean seeds was correlated with an absence of proanthocyanidin content in their seed coats, but their impact on hormone-related processes is not known. A putative gibberellic acid-regulated protein gene, *Phvul.001G006300,* was negatively associated with proanthocyanidin accumulation in cranberry beans (Fig. 8d). Interestingly, this gene was upregulated in the non-darkening RIL (Additional file 3). Gibberellic acids are plant hormones with numerous biological roles in the plant, including the activation of starch breakdown enzymes in embryonic seed tissues leading to a release from dormancy [62]. Furthermore, seed coat growth is linked to accumulation of bioactive gibberellic acids, specifically 13-hydroxylated gibberellic acids in these tissues during pea seed maturation [63]. *Phvul.001G006300* was one of 67 genes belonging to cluster 4, which included hormone-related genes that were more upregulated in non-darkening than darkening RIL seed coats (see Additional file 5). Biochemical and functional characterization studies of the proteins encoded by these hormone-related genes are required to better understand their respective relevance for seed coat development in non-darkening cranberry beans. As the non-darkening cranberry beans are proanthocyanidin deficient, the possibility remains that hormonal regulation of the dormancy period is varied from that operating in darkening cranberry beans.

## Conclusions

Seed coat darkening in dicotyledonous species is dependent upon proanthocyanidin oxidation to reactive quinones [7, 15–17]. Interestingly, this phenomenon is apparent in genotypes with a ready availability of seed coat proanthocyanidins, including the postharvest darkening susceptible cranberry bean RIL germplasm investigated in this study. Moreover, research on the model plant organisms Arabidopsis and *M. truncatula* has established that proanthocyanidin levels in the seed coat are associated with a fully functional biosynthetic pathway [23–25, 31–33]. An RNA-seq analysis revealed that nearly 5% of all seed coat genes were differentially expressed between a darkening- and a non-darkening cranberry bean RIL, which is consistent with the transcriptomic analysis of seed coats from diversely coloured *B. juncea* seeds [53]. All proanthocyanidin biosynthesis genes (including *PvLAR* and *PvANR1*) were co-ordinately upregulated in the darkening RIL, and their seed developmental profiles were consistent with the expression of *PvMYB*s. These phenomena were largely absent in non-darkening cranberry beans. Notably, proanthocyanidin accumulation in seed coats of the darkening susceptible RIL was highly associated with the upregulated expression of three proanthocyanidin biosynthesis genes, *PvF3H1* , *PvDFR1*, and *PvANR1*. Like the majority of ANRs characterized to date [29, 55–59], *Pv*ANR1 activity was NADPH-dependent and catalyzed the formation of epicatechin and catechin from cyanidin. All three of these phenolic compounds are evident in seed coats of darkening cranberry beans, but absent in non-darkening seeds [5, 41]. Interestingly, *Pv*ANR1 activity was inhibited by high concentrations of cyanidin. Together the findings in this study suggest that: (i) proanthocyanidin accumulation in cranberry bean seed coats is linked to transcriptional regulation of the proanthocyanidin pathway; (ii) *Pv*ANR1 serves as the major enzyme for proanthocyanidin formation; and (iii) substrate inhibition of this activity could represent an in vivo control mechanism for limiting proanthocyanidin accumulation. The combined transcriptomic and biochemical information given here is of critical importance for future breeding strategies aimed at limiting darkening in *P. vulgaris* seeds.

## Methods

### Chemicals and plant material

Unless otherwise mentioned, chemicals were purchased from Sigma-Aldrich (Oakville, Ontario, Canada). Darkening and non-darkening cranberry bean RILs were created by the Bean Breeding Program at the University of Guelph (Guelph, Ontario, Canada) from a cross between a parental line, ‘Etna’, that is susceptible to postharvest darkening [41] and ‘Wit-rood boontje’, a cranberry-like bean parental line obtained from the USDA National Center for Genetic Resources Preservation at Ft. Collins, CO (GRIN Accession number: PI 439540) that does not undergo postharvest-related darkening [6], and herein is referred to as non-darkening. The ‘Etna’ parental line was obtained from Seminis Vegetable Seeds. Inc. (Woodland, California, USA). Briefly, crosses between the parents were made in a growth room at the University of Guelph. The F_1_ and F_2_ seeds were allowed to self and the F_3_ seeds were screened for their reaction to ultraviolet C light [8] to identify lines that were darkening and non-darkening. The lines were selfed for additional generations to produce darkening and non-darkening recombinant inbred lines.

On September 11, 2012, 135 seeds of a non-darkening RIL and 135 seeds of a darkening RIL from the aforementioned cross (F_5_ progeny) were sown in 1.5 L pots (one seed per pot) containing Sunshine Mix #2 / LB2 soil (SunGro Horticulture, St. Catharines, Ontario, Canada) saturated with a water soluble fertilizer (N:P:K, 20:8:20) (Plant Products, Ancaster, Ontario, Canada) at 1.25 g L^−1^. In each of three separate greenhouses at the University of Guelph, 45 darkening and 45 non-darkening RIL cranberry bean plants were cultivated in a completely randomized design under constant day and night temperatures of 26 and 16 °C, respectively. Supplemental high pressure sodium lighting was supplied between 06:00 and 22:00 h when the incident sunlight was less than 200 watts m^−2^. Plants were irrigated daily with the aforementioned fertilizer mixture for 30 min until the last day of seed pod harvesting (November 28, 2012). In each greenhouse, seed pods of both RIL plants were harvested at early, intermediate and mature stages of development, which corresponded to pod lengths of 9–15 cm, 15–20 cm, and 20–28 cm, respectively. For each greenhouse replicate, RIL-specific seed pods were pooled from 45 plants at each developmental stage. Thereafter, seeds were removed from pods and seed coats were manually decorticated and frozen in liquid N_2_. The frozen seed coat material was powdered with a mortar and pestle under liquid N_2_, and stored at −80 °C until required for proanthocyanidin and transcript analyses. For both RILs, the remainder of the harvested mature seeds were stored in sealed plastic bags at 4 °C for up to 48 months.

### DMACA staining

In order to visualize proanthocyanidin accumulation in whole seeds, aged seeds of both RILs were subjected to DMACA staining, using a previously described method with the following modifications [37]. Briefly, seeds previously stored at 4 °C were transferred to ambient temperature and soaked in water for 24 h. Thereafter, the imbibed seeds were immersed in a solution of ethanol containing 0.8% (*w*/*v*) HCl and 0.5% (*w*/*v*) DMACA for 60 min, followed by washing in 70% (*v*/v) ethanol for 60 min.

### Proanthocyanidin extraction and quantification

For each biological replicate, frozen cranberry bean seed coat powder (1.5 g) was extracted with 10 volumes of acetone: MilliQ-processed water (13:7, *v*/v) as described previously [64], by pulsing the suspension ten separate times for 30 s with a sonic dismembrator set to 80% of the maximum amplitude (Thermo Fisher Scientific, Mississauga, Ontario, Canada). Pauses of 30 s were used between successive pulses. Thereafter, tissue extracts were rotated on an orbital shaker (Adams™ Nutator; Becton, Dickinson and Company, Franklin Lakes, New Jersey, USA) for 2 h at 24 °C, and pelleted at 2500 x *g* for 10 min at 24 °C. Aliquots (70 μL) of the supernatants were transferred to microplate wells and combined with DMACA colorimetric assay reagent to final volumes of 280 μL. Proanthocyanidin levels were detected at 640 nm, as described previously [44, 64], using a SpectraMax Plus 384 Microplate Reader (Molecular Devices, Sunnyvale, California, USA) and compared to known amounts (0.34 to 2.02 μg) of an authentic procyanidin A2 standard (Extrasynthese, Genay, France). For each biological replicate, proanthocyanidin determinations were performed in triplicate. One-way analysis of variance in SAS 9.3 (SAS Institute Inc., Cary, North Carolina, USA) was used to analyse the total proanthocyanidin data at the α = 0.05 level.

### RNA preparation and sequencing

High-quality total RNA was isolated from cranberry bean RIL seed coats following a modified procedure for the exclusion of polyphenolic compounds [65]. Briefly, frozen pulverized seed coat powder (500 mg) samples were homogenized with 3 mL of 100 mM Tris-HCl (pH 7.5) containing 2% (*w*/*v*) hexadecyltrimethylammonium bromide detergent, 2% (*w*/*v*) polyvinylpyrrolidone (average molecular weight of 40,000 g mol^−1^), 25 mM ethylenediaminetetraacetic acid, 2 M NaCl, 2% (*v*/v) β-mercaptoethanol and 0.5 g L^−1^ spermidine, and incubated at 65 °C for 10 min. The samples were inverted periodically during the incubation period. The cell residues were pelleted by centrifugation at 10000 x *g* for 30 min at 4 °C, and the aqueous phases were combined with equal volumes of chloroform and re-centrifuged, as described previously. The aqueous phases were combined with 2 M LiCl and total RNA samples were precipitated for 18 h at 4 °C. Thereafter, the RNA samples were pelleted by centrifugation at 20000 x *g* for 15 min at 4 °C, and washed with ice-cold 70% (*v*/v) ethanol. RNA was quantified with a NanoDrop 1000 UV/Vis spectrophotometer (NanoDrop Technologies, Wilmington, Delaware, USA) and analyzed for quality and integrity with standard molecular biology techniques [66].

For each greenhouse/developmental stage replicate, RNA preparations were depleted of rRNA with an Illumina Ribo-Zero magnetic kit (Mandel Scientific Company Inc., Guelph, Ontario, Canada,), and verified for the absence of rRNA contaminants with the Agilent RNA 6000 Pico Kit (Agilent Technologies, Mississauga, Ontario, Canada) on an Agilent 2100 Bioanalyzer as per the manufacturers’ instructions. Preparation of cDNA libraries and next generation sequencing was performed at The Centre for Applied Genomics, Hospital for Sick Children (Toronto, Ontario, Canada). Briefly, for each sample 400 ng of mRNA was used for library preparation with the Illumina TrueSeq RNA sample preparation kit v2. The cDNA libraries were subsequently sequenced in two lanes of Illumina HiSeq 2500 platform to generate paired-end reads of 101 bp.

### Seed coat transcriptome assembly and analysis

The paired sequence reads were trimmed for adapter removal with FASTQ Quality Trimmer [67] to a minimum of 80% of the original sequence length, poor quality reads were eliminated using a minimum Phred score of 32. For each seed coat cDNA library, the Illumina sequence reads (in FASTQ format) were mapped to the genomic sequence of the *P. vulgaris* G19833 reference genome (assembly version 1.0; [68, 69]) with Bowtie2 using default parameters, including a maximum sum of mismatch qualities across the alignment of 70. The data was analyzed for exon-exon junctions in TopHat as described previously [70]. Transcriptome assemblies were generated in Cufflinks, and annotation was performed with Cuffcompare. Differentially expressed genes were identified with Cuffdiff, and transcript abundance was reported as FPKM, using cummeRbund in R [71].

A cluster analysis was performed to identify genes with similar expression patterns in the seed coat transcriptome. To this end, raw read counts for all differentially expressed genes were obtained from Binary Alignment/Map (BAM) files using samtools [72] v0.1.17 and HTSeq v0.6.1p2 [73]. Clustering of genes was performed with the HTSCluster v2.0 package [74] in R [71] with the number of clusters ranging from 1 to 50. A model containing 14 clusters was selected a posteriori using the model selection criterion Dimension jump [75]. Thereafter, GO enrichment analysis was performed on the gene cluster model conducted using the Singular Enrichment Analysis tool available on AgriGO v1.0 [76] with a significance level of 5% using Fisher statistical testing and Yekutieli multi-test adjustment.

A TFBS enrichment analysis was performed for all differentially expressed genes. To this end, we downloaded the *Phaseolus vulgaris* genome assembly (Pvulgaris_218_v1.0.fa) and its annotation (Pvulgaris_218_v1.0.gene.gff3) from Phytozome [68, 69]. All scaffolds were removed from the genome assembly, and chromosomal sequences were retained. To investigate groups of genes for transcription factor binding sites, gene start positions were isolated from the .gff3 file. Differentially expressed genes with no annotated sequence in the bean genome were excluded from the analysis. For each gene group, we extracted sequence 500 bp upstream from each transcription start site, excluded Ns (and nucleotides upstream of Ns), and searched the sequence and its reverse complement for one or more motif binding sites. The analysis searched the following sites: C[AGCT]GTT[AG] and CACGTG, where [AGCT] indicates any single nucleotide, and quantified the number of genes within each group of differentially expressed genes with at least one binding site. All analyses were performed with custom perl scripts.

PCA was performed in R [71] to determine whether there was an association between RNA-seq transcript profiles and proanthocyanidin accumulation patterns in cranberry bean seed coats. In order to generate scores for the PCA, transcript levels of the differentially expressed genes (expressed as FPKM) corresponding to each of the 18 seed coat replicates were converted to uncorrelated variables using an orthogonal linear transformation. Thereafter, the components accounting for 95% of the cumulative variance were considered for the correlation analysis. A correlation analysis was performed between the selected PCs and the seed coat total extractable proanthocyanidin levels in R. A score plot was generated for the PCs that were highly correlated with seed coat proanthocyanidin levels. Finally, transcripts with the highest contribution for each of these PCs were identified with a loading plot analysis.

### Cloning, expression and purification of recombinant *Pv*ANR1

High-quality total RNA was extracted from seed coats of developing cranberry beans harvested from darkening RIL plants as described above, and assessed for quality and integrity using standard molecular biology methods [66]. Following DNase I treatment, a first strand cDNA library was prepared from 2.5 μg total RNA using the SuperScript® First-Strand Synthesis System (Invitrogen Life Technologies, Burlington, Ontario, Canada) according to the manufacturer’s protocol. Forward (5′ C ATG GCC ACT GTC AAG AAA ATT GGA AAG 3′) and reverse (3′ GCA TAA CAA TTT CCA AAT TCA GTT CTT GAG 5′) oligonucleotide primers were used to amplify the *PvANR1* open reading frame from cDNA with standard techniques [66]. PCR was performed with the Platinum Taq DNA Polymerase High Fidelity enzyme (Invitrogen Life Technologies) under the following conditions: initial denaturation step of 1 min at 94 °C followed by 25 cycles of 94 °C for 30 s, 55 °C for 30 s, and 68 °C for 1 min, and a final extension step at 68 °C for 10 min. Thereafter, the amplified PCR product was analyzed by agarose gel electrophoresis. A 1014 bp PCR product was gel purified using a GeneJET Gel Extraction Kit (Thermo Fisher Scientific) and ligated into pGEM-T subcloning vector (Promega Corporation, Madison, Wisconsin, USA). The pGEM-T-*PvANR1* construct was digested with *Nco*I and *Not*I, and ligated into the corresponding restriction sites of pET-30b vector, in order to generate an N-terminal His_6_-tagged *Pv*ANR1 with a cleavable enterokinase linker. The pET-30b-*PvANR1* construct was confirmed by sequencing and transformed into *E. coli* BL21 competent cells (kindly provided by Dr. Barry J. Shelp, Department of Plant Agriculture, University of Guelph; originally attained from EMD Millipore, Etobicoke, Ontario, Canada). Thereafter, *E. coli* pET-30b-*PvANR1* transformants were cultured on Luria-Bertani media supplemented with kanamycin (50 μg mL^−1^) at 37 °C under continuous shaking (180 rpm) until the A_600_ reached the mid-logarithmic growth phase. Cultures (6 L) were induced with 400 μM isopropyl β-D-thiogalactopyranoside and shaken at 180 rpm for 3 h at 20 °C. Cells were pelleted by centrifugation at 3500 x *g* for 10 min at 4 °C, flash-frozen in liquid N_2_ and stored at −80 °C (for a maximum of 5 days) until required for protein purification.

All protein extraction steps were performed at 4 °C. The frozen bacterial cells were resuspended in 200 mL of protein extraction buffer containing 20 mM sodium phosphate (pH 7.5), 500 mM NaCl, 10 mM imidazole, 10 mM β-mercaptoethanol, 10% (*v*/v) glycerol, 1 mM phenylmethanesulfonyl fluoride and 1 X Sigma Protease Inhibitor Cocktail. The resuspended cells were sonicated for 10 min (30 s pulses at 30% of maximum amplitude with 30 s intervals) using a sonic dismembrator (Thermo Fisher Scientific). The cell lysate was centrifuged at 19000 x *g* for 15 min and the supernatant was passed through a 0.45 μm polyvinylidene difluoride membrane filter (Millex-HV; EMD Millipore). The clarified supernatant was applied at 1 mL min^−1^ onto a 1 mL HisTrap™ HP column (GE Healthcare Life Sciences; Mississauga, Ontario, Canada) pre-equilibrated with buffer A (20 mM sodium phosphate pH 7.5, 500 mM NaCl and 10 mM imidazole) and coupled to an ÄKTA FPLC system. The unbound proteins were removed from the column by washing with 20 column volumes of Buffer A. The recombinant His_6_-*Pv*ANR1 was eluted with a linear gradient of 10–500 mM imidazole in buffer A (8 mL, fraction size = 2 mL). Fractions containing a major A_280_ peak were pooled and passed through a PD-10 Sephadex™ G-25 gel filtration column (GE Healthcare Life Sciences) pre-equilibrated with enterokinase reaction buffer (20 mM Tris-HCl, pH 8.0; 50 mM NaCl; and 2 mM CaCl_2_). Enterokinase cleavage of the His_6_-tag from the recombinant *Pv*ANR1 preparation was performed with enterokinase light chain (2 μg mL^−1^) as per the manufacturer’s protocol (New England BioLabs, Whitby, Ontario, Canada). Modifications to the protocol included incubating the His_6_-tagged *Pv*ANR1 with 4 ng of enterokinase for 30 min at 25 °C. The cleaved protein was purified by application on a PD-10 column pre-equilibrated with buffer B (20 mM sodium phosphate, pH 7.5; and 500 mM NaCl) followed by a His-Select Nickel Affinity column pre-equilibrated with buffer B. The His_6_-tag free *Pv*ANR1 preparation was concentrated in an Amicon Ultra-15 Centrifugal Filter Device with a 10 kDa cut-off as per the manufacturer’s instructions (EMD Millipore).

Protein concentrations were determined with the Bradford method [77] via the Bio-Rad Protein Assay kit (Bio-Rad Laboratories, Mississauga, Ontario, Canada) and compared to known amounts of an authentic bovine γ-globulin standard. The final *Pv*ANR1 concentration was adjusted to 1 mg mL^−1^ in buffer B containing 20% glycerol (*v*/v), divided into 200 μL aliquots, flash-frozen and stored at −80 °C prior to their use in enzymatic assays. The recombinant *Pv*ANR1 preparation was evaluated for purity and integrity by sodium dodecyl sulfate polyacrylamide gel electrophoresis (SDS-PAGE) using 10% (*w*/*v*) acrylamide gels according to a previously published protocol [78]. The removal of the His_6_-tag from *Pv*ANR1 was assessed by immunoblotting. To this end, SDS-PAGE-gels were transferred to a 0.45 μm polyvinylidene difluoride membrane (EMD Millipore) using standard procedures [79], immunoblots were probed with an anti-His_6_-tag primary antibody (1:2000 dilution; Santa Cruz Biotechnology, Dallas, Texas, USA), followed by a secondary antibody conjugated to the enzyme alkaline phosphatase (1:30,000 dilution; Sigma-Aldrich). Immunoreactive bands were detected with a chromogenic alkaline phosphatase substrate reagent kit (Bio-Rad Laboratories) according to the manufacturer’s instructions.

### In vitro assay of recombinant *Pv*ANR1 activity

Recombinant *Pv*ANR1 activity was assayed in vitro at 30 °C in a final volume of 400 μL. *Pv*ANR1 activity was directly proportional to the amount of recombinant enzyme added (within the 10–90 μg range) to the assay mixture and time (2 to10 min). Unless otherwise mentioned, assays included 50 mM 4-(2-hydroxyethyl)piperazine-1-ethanesulfonic acid (pH 7.0), 800 μM NADPH, 100 μM cyanidin chloride (Extrasynthese), 50 μg recombinant *Pv*ANR1, and 5% (*v*/v) methanol. Cyanidin chloride was freshly prepared in 96% (*v*/v) methanol containing 0.4 mM of methane sulfonate buffer (pH 2.0), and reactions were initiated by their addition to the assay mixture and incubated for 10 min at 30 °C. All assays were terminated by the addition of ethyl acetate (500 μL), and reaction products were partitioned into the organic phase by vortexing for 30 s and centrifugation at 18800 x *g* for 1 min. Upon removal of the organic phase, the aqueous phase was re-extracted with ethyl acetate as described above. The organic layers from the successive extractions were pooled and dried under a stream of argon gas at room temperature. As a control, assays were performed in the absence of recombinant *Pv*ANR1.

The dried reaction residue was resuspended in 100 μL of methanol, filtered with 0.45 μm polytetrafluoroethylene syringe filter (Mandel Scientific Company Inc.), and 5 μL injections were separated on a Kinetex pentafluorophenyl column (100 × 4.6 mm, 2.6 μm Phenomenex, Torrence, California, USA) coupled to an Agilent 1200 HPLC-DAD system. The reaction products were eluted with a gradient of solvent B (CH_3_CN:C_2_HF_3_O_2_, 99.9:0.1, *v*/v) in solvent A (H_2_O:CH_3_CN:C_2_HF_3_O_2_, 90:9.9:0.1, *v*/v/v) of 0–25%, 0–10 min; 25–81.8%, 10–20 min; and 81.8–100%, 20–25 min at a flow rate of 0.8 mL min^−1^. In assays containing cyanidin, peaks corresponding to catechin (retention time = 3.9 min) and epicatechin (retention time = 4.7 min) were detected at 280 nm. In either case, retention times and UV spectra were compared to known amounts of authentic (+)-catechin and (−)-epicatechin standards (both from Extrasynthese). Kinetic parameters were estimated using non-linear regression models available in SigmaPlot (version 12.3) Enzyme Kinetics Module.

### Seed germination analysis

Seed germination assays were performed essentially as described previously [60]. Aged seeds (previously stored at 4 °C for 48 months) of both cranberry bean RILs were washed with 70% (*v*/v) ethanol for 2 min, followed by three successive rinses with sterile water (1 min each). Seeds were dried for 15 min in a sterile cabinet. For each RIL, 104 seeds were sown on 13 plates containing agar medium, and then these were incubated at 25 °C under darkness for 9 days in an environment-controlled chamber. Seeds were analyzed daily for evidence of radicle emergence, the first physical sign of germination. This experiment was performed in triplicate. For statistical comparison of seed germination percentages across both RILs, a one-way analysis of variance was performed with SAS v 9.4 (proc mixed method) and means were compared with the least significant difference method at α = 0.05 level.

## Additional files

Additional file 1: Comprehensive list of gene expression values (in FPKM) 18 seed libraries representing darkening and non-darkening RILs. (XLSX 24083 kb)

Additional file 2: List of genes upregulated in seed coats of darkening relative to non-darkening cranberry bean RILs as a function of seed development. (XLSX 72 kb)

Additional file 3: List of genes upregulated in seed coats of non-darkening relative to darkening cranberry bean RILs as a function of seed development. (XLSX 48 kb)

Additional file 4: List of genes upregulated in seed coats of both non-darkening and darkening cranberry bean RILs at different stages of seed development. (XLSX 14 kb)

Additional file 5: Differentially expressed genes belonging to cluster 1 to 14 and their respective seed coat transcript abundance levels at each developmental stage for darkening and non-darkening cranberry beans RILs. (XLSX 314 kb)

Additional file 6: Figure S1. Expression patterns of unknown genes highly associated with proanthocyanidin accumulation in cranberry bean seed coats. **Figure S2.** Phylogenetic comparison of *P. vulgaris* ANR amino acid sequences with known ANRs from other plant species. (DOCX 78 kb)

## Abbreviations

ANR: Anthocyanidin reductase
ANS: Anthocyanidin synthase
bHLH: Basic helix-loop-helix
DFR: Dihydroflavonol 4-reductase
DMACA: 4-Dimethylaminocinnamadehyde
F3H: Flavanone 3-hydroxylase
F3′H: Flavonoid 3′-hydroxylase
FPKM: Fragments per kilobase of exon per million fragments mapped
GO: Gene ontology
His_6_: Hexahistidine
IMAC: Immobilized metal affinity chromatography
LAR: Leucoanthocyanidin reductase
MATE: Multidrug and toxic extrusion
MYB: Myeloblastosis proto-oncogene
PCA: Principal component analysis
RIL: Recombinant inbred line
RNA-seq: RNA sequencing
TFBS: Transcription factor binding site
TT: Transparent testa
