# Supplementary material for: Proanthocyanidin accumulation and transcriptional responses in the seed coat of cranberry beans (Phaseolus vulgaris L.) with different susceptibility to postharvest darkening
Source: BMC Plant Biol. 2017 May 25;17:89. doi: 10.1186/s12870-017-1037-z (PMC5445279; doi:10.1186/s12870-017-1037-z)
Supplement: Supplementary file 6 — Expression patterns of unknown genes highly associated with proanthocyanidin accumulation in cranberry bean seed coats. Figure S2. Phylogenetic comparison of P. vulgaris ANR amino acid sequences with known ANRs from other plant species. (DOCX 78 kb) [file 12870_2017_1037_MOESM6_ESM.docx]

**Additional File 6**

**Figure S1.** **Expression patterns of unknown genes in seed coats of darkening and non-darkening cranberry bean RILs sampled at early (E), intermediate (I) and mature (M) stages of seed development.** Transcript levels for each gene at each stage are expressed as FPKM. Each datum represents the mean ± standard error of three greenhouse replicates.

Statistical differences were established based on a one-way ANOVA with Tukey’s test for the multiple mean comparisons. Shared letters indicate no significant differences at p≤.05.


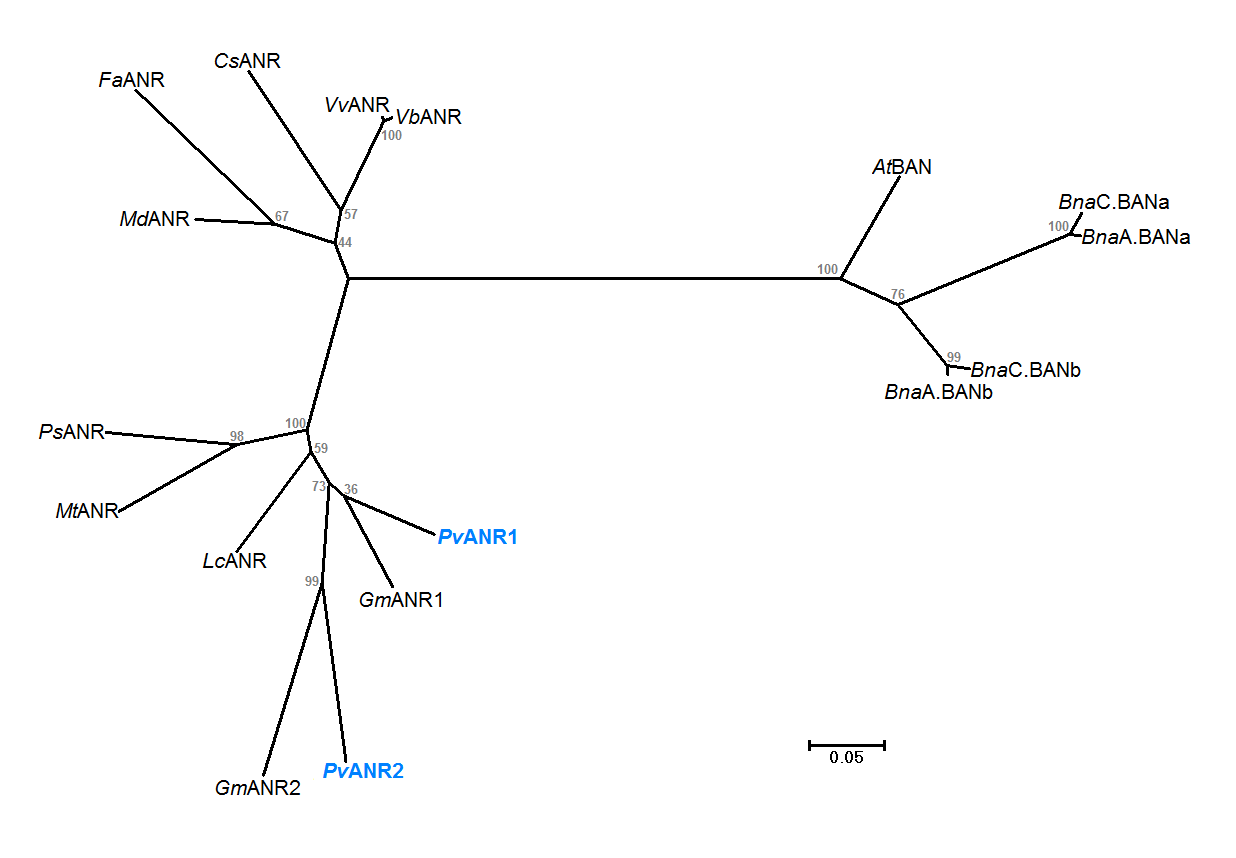


**Figure S2. Phylogenetic comparison of *P. vulgaris* ANR amino acid sequences with known ANRs from other plant species.**  *In silico translations* of *P. vulgaris* ANR coding sequences were aligned to amino acid sequences of other plant ANRs using ClustalW (www.genome.jp/tools/clustalw; Larkin et al., 2007); thereafter, the maximum likelihood method in MEGA 6.06 was used to construct the unrooted tree (Tamura et al., 2013). Numbers proximal to each node represent the percent support values from the bootstrap analysis using 500 iterations. GenBank™ accession numbers for each ANR are provided in parentheses: *Arabidopsis thaliana* BANYULS, *At*BAN (AAF23859.1); *Brassica napus* A.BANa, *Bna*A.BANa (ACY30423.1); *Bna*A.BANb (ACY30424.1); *Bna*C.BANa (ACY30421.1); *Bna*C.BANb (ACY30422.1); *Camellia sinensis* ANR, *Cs*ANR (ADF43751.1); *Fragaria* x *ananassa* ANR, *Fa*ANR (ABG76842.1); *Glycine max* ANR1, *Gm*ANR1 (AEM23932.1); *Gm*ANR2 (AEM45797.1); *Lotus corniculatus* ANR, *Lc*ANR (ABC71335.1); *Malus domestica* ANR, *Md*ANR (AEL79861.1); *Medicago truncatula* ANR, *Mt*ANR (AAN77735.1); *Pisum sativum* ANR, *Ps*ANR (AII26022.1); *Vitis bellula* ANR, *Vb*ANR (AFG28175.1); *Vitis vinifera* ANR, *Vv*ANR (BAD89742.1). For *in silico* translations, GenBank™ accession numbers corresponding to cranberry bean ANR complete coding sequences are provided in parantheses: *Phaseolus vulgaris ANR1*, *PvANR1*  (KY084241); *Phaseolus vulgaris ANR2*, *PvANR2* (KY084242). The scale bar represents 0.05 amino acid substitutions per site.

**References:**

Larkin MA, Blackshields G, Brown NP, Chenna R, McGettigan PA, McWilliam

H, Valentin F, Wallace IM, Wilm A, Lopez R, Thompson JD, Gibson TJ,

Higgins DG. Clustal W and Clustal X version 2.0. Bioinformatics. 2007; 23:

2947–2948.

Tamura K, Stecher G, Peterson D, Filipski A, Kumar S. MEGA6: molecular

evolutionary genetics analysis version 6.0. Mol. Biol Evol. 2013; 30: 2725–2729.
